# Can Data Science Inform Environmental Justice and Community Risk Screening for Type 2 Diabetes?

**DOI:** 10.1371/journal.pone.0121855

**Published:** 2015-04-14

**Authors:** J. Allen Davis, Lyle D. Burgoon

**Affiliations:** National Center for Environmental Assessment, Office of Research and Development, United States Environmental Protection Agency, Research Triangle Park, North Carolina, United States of America; University of Michigan Medical School, UNITED STATES

## Abstract

**Background:**

Having the ability to scan the entire country for potential “hotspots” with increased risk of developing chronic diseases due to various environmental, demographic, and genetic susceptibility factors may inform risk management decisions and enable better environmental public health policies.

**Objectives:**

Develop an approach for community-level risk screening focused on identifying potential genetic susceptibility hotpots.

**Methods:**

Our approach combines analyses of phenotype-genotype data, genetic prevalence of single nucleotide polymorphisms, and census/geographic information to estimate census tract-level population attributable risks among various ethnicities and total population for the state of California.

**Results:**

We estimate that the rs13266634 single nucleotide polymorphism, a type 2 diabetes susceptibility genotype, has a genetic prevalence of 56.3%, 47.4% and 37.0% in Mexican Mestizo, Caucasian, and Asian populations. Looking at the top quintile for total population attributable risk, 16 California counties have greater than 25% of their population living in hotspots of genetic susceptibility for developing type 2 diabetes due to this single genotypic susceptibility factor.

**Conclusions:**

This study identified counties in California where large portions of the population may bear additional type 2 diabetes risk due to increased genetic prevalence of a susceptibility genotype. This type of screening can easily be extended to include information on environmental contaminants of interest and other related diseases, and potentially enables the rapid identification of potential environmental justice communities. Other potential uses of this approach include problem formulation in support of risk assessments, land use planning, and prioritization of site cleanup and remediation actions.

## Introduction

Communities and public health agencies, such as the US Environmental Protection Agency (EPA), would benefit from being able to quickly screen local communities, and potentially the entire country, for possible geographic “hotspots” for increased risk of developing chronic diseases due to varied socioeconomic, demographic, genetic, and environmental factors. Leveraging data science approaches (i.e., extracting knowledge from multiple, disparate sources of data) should allow for the identification of these geographic areas whose populations are at increased risk due to multiple risk factors.

Emerging evidence suggests that race, socioeconomic factors, and where one lives may adversely impact one’s risk of developing type 2 diabetes mellitus (T2DM) [1,2]. Recent epidemiologic evidence regarding the association of T2DM and environmental contaminants additionally suggests a relationship between some heavy metals (arsenic) and persistent organic pollutants (PCBs, *p*,*p’*-DDE) [3]. However, the current evidence fails to support associations with other contaminants such as mercury and cadmium [3]. Identified gaps in current epidemiologic database include how co-exposures, comorbidities, and genetic variants modify the association between T2DM and individual environmental pollutants. As genomic determinants of T2DM have been shown to demonstrate heterogeneity across populations [4], it is possible that these differential genetic susceptibilities may interact with environmental factors to tip the scale in favor of developing T2DM. Therefore, information regarding the prevalence of genetic polymorphisms conferring increased risks of developing T2DM within various human populations would allow for the identification of potentially susceptible populations. Finally, the locations where these susceptible populations live can be identified through the use of census data, and geographic information systems (GIS) can be used to generate maps to display those hotspots of genetic risk. As a pilot study, this analysis focused on the state of California and the characterization of the risk of developing T2DM in three separate ethnic groups due to a single nucleotide polymorphism (SNP).

## Materials and Methods

### Genetic Data Mining

We performed literature and database searches to identify several genotypes that have been shown to be associated with T2DM [5]. As a pilot study, we decided to focus on the C/T rs13266634 polymorphism in the solute carrier family 30 member 8 (SLC30A8) zinc transporter for our study based on its role in insulin packaging. Zinc has been associated with insulin biosynthesis [6], and chronic decreased zinc intake has been associated with an increased risk of diabetes [7]. Specifically, the SCL30A8 Zn transporter is expressed in the pancreatic beta-cell secretory vesicles, and is primarily responsible for transporting Zn from the cytoplasm into the secretory vesicles for insulin maturation, storage, and secretion [8]. The SNP rs13266634 has been shown to be associated with T2DM in multiple populations [9–17]. The risk allele in rs13266634 is C, while the minor allele is T [10,18]. As SCL30A8 requires Zn for its catalytic function, it is particularly susceptible to competition from other divalent cations. Therefore, characterizing the differential risk this polymorphism confers on individual populations may help refine the determination of any association between T2DM and exposure to divalent heavy metals in the environment. We used the Database of Single Nucleotide Polymorphisms (dbSNP) [19] and subsequent literature searching to identify the prevalence of this SNP in various human populations.

### Calculation of Population Attributable Risk

Individual studies reporting increased odds of T2DM in Asian or European carriers of the rs13266634 polymorphism were identified from a previously published 2011 meta-analysis [8]. For this analysis, homozygous carriers of the risk allele (i.e., CC) were considered to be at greatest risk of developing T2DM compared to dominant carriers (i.e., CT and TT). Using information provided [8], studies were identified for inclusion if they provided enough information to calculate the total frequency of the CC genotype in the study population (i.e., either the actual genotype numbers for cases and controls or the risk allele frequency). If a study did not explicitly report genotype numbers for cases and controls, that study was still included in the analysis if the risk allele frequency was reported in the control and case populations. Assuming Hardy-Weinburg equilibrium in these populations, the expected number of carriers of the CC genotype can be calculated as follows:

$${\text{Expected CC=p}}^{\text{2}}\text{n}$$ (1)

where *p* is the reported risk allele frequency, and *n* is the case and control study populations. Study specific frequencies of the CC genotype were calculated by summing the number of case and control carriers of the CC genotype and dividing by the total study population. Weighted CC genotype frequencies for Asian or European populations were then calculated using individual study sizes for the weights. In order to characterize the genetic risk the rs13266634 polymorphism confers to homozygous carriers, the population attributable risk (PAR) for each ethnicity was calculated as follows [20,21]:

$$\text{PAR=100 }\times \frac{\left(\text{E}\times \left(\text{OR-1}\right)\right)}{\text{(1+}\left(\text{E}\times \left(\text{OR-1}\right)\right)}$$ (2)

where *E* is the frequency of the CC genotype (calculated as described above) and *OR* is the reported odds ratio for developing T2DM in the study populations. The PAR is the proportion of T2DM cases in the various populations expected to occur solely due to the presence of the CC risk genotype. Pooled odds ratios and 95% confidence limits were calculated from individual studies as described previously [8]. Assuming a dominant model (i.e., CC vs. [CT + TT]), a fixed-effect model was used to calculate a pooled OR across all included Asian and European study populations. The pooled OR was considered statistically significant with Z-test p value < 0.05. To determine whether using a fixed-effect model was appropriate, the heterogeneity of the individual studies was assessed using the Χ^2^-based Q test. Evidence of statistical heterogeneity was assumed if the p-value for the Q test was < 0.10, or the I^2^ value was > 50%. If evidence of heterogeneity was evident, a random-effects model was then used. The CC frequency and PAR for a single Mexican Mestizo population was calculated using information published in the available literature [22]. All statistical analyses were conducted using the R statistical package (version 3.0.1, the R Foundation for Statistical Computing).

### Generating Hotspot Maps for California

The following demographic data for California on the census tract level were extracted from the 2007–2011 American Communities Survey: Caucasian population, Asian population, and Mexican (of any race) population [23]. Total population was calculated as the sum of the Caucasian, Asian-American, and Mexican populations (i.e., the “total” population for this analysis excludes population figures for any other ethnicities). For the purposes of identifying potential hotspots of T2DM incidence due to the rs1326634 polymorphism, the PARs calculated for European, Asian, and Mexican populations [8,22] were assumed to be representative of the Caucasian, Asian, and Mexican (of any race) populations reported in the census data. The census tract level demographic data were joined to a census tract shapefile for California using ArcGIS (version 10.1). A weighted average PAR for the total population in each census tract was then calculated using the subgroup populations as the weights. By extension, subgroup-specific PARs for each census tract can be calculated by multiplying the population of that subgroup by the subgroup-specific PAR and then dividing by the total population; these values represent the proportion of T2DM cases expected to occur in the total population solely due to the presence of the risk allele in that specific subgroup. Shaded maps were constructed displaying the PAR (categorized by quintile) in each Californian census tract due to Caucasian, Asian-American, or Mexican-American populations individually, or in aggregate. Finally, “hotspots” of genetic T2DM susceptibility were identified by determining which counties in California had >25% of their population (Caucasian, Asian-American, or Mexican-American only) residing in census tracts in the highest quintile for Total PAR. All maps were created using ArcGIS software by ESRI. ArcGIS and ArcMap are the intellectual property of ESRI and are used herein under license (Copyright ESRI, all rights reserved).

## Results

Of the studies previously identified [8], 22 studies (28 individual study populations, herein referred to as “cohorts”) were included in the current analysis [10,11,13,16,24–41]. After careful consideration, the following cohorts were excluded from the analysis: 3 cohorts investigated non-European or Asian populations (Ashkenazi Jews and Pima Indians) [24,42,43], 2 cohorts utilized a non-case-control study design [44,45], and 4 cohorts provided inadequate information to calculate genotype frequencies (see Methods) [9,12,17,46]. Cohorts of African populations [16,24,47] were excluded from this analysis as the pooled OR for those cohorts was not statistically significantly increased (1.20 [0.90–1.40]) [8].

Initial prevalence information was obtained from the dbSNP database, which contains the HapMap data. The rs13266634 SNP has an estimated risk allele frequency in the Mexican population of 81% (CC/CT). In the Caucasian and Asian populations, the risk allele has an estimated prevalence of 73.6% and 55.6%, respectively. For the studies used in the pooled cohort OR and PAR calculations, average risk allele frequencies in T2DM cases for Caucasians (70.4%) and Asians (61.7%) were similar to those reported in the dbSNP database; the risk allele frequency in the Mexican Mestizo population included in this analysis [22] also was similar the reported value in the database. When a weighted CC genotype frequency was calculated for these three ethnicities, Mexican Mestizos had the highest CC frequency (56.3%), followed by Caucasians (47.4%) and Asians (37.0%) (Table 1).

**Table 1:** CC genotype frequencies for T2DM cases and controls, with calculated population attributable risks.

Cohort	Cases (N)	Cases—CC Genotype	Cases RAF	Controls (N)	Controls—CC Genotype	Controls RAF	Total N	Total CC	Frequency CC Genotype	Weighted Frequency CC	PAR^a^
**Asian Cohorts**											
Horikoshi	860	328	0.604^b^	859	293	0.57^b^	1719	621	0.361	0.370	0.065
Steinsthosdottir	1426	464	0.566	970	259	0.523	2396	723	0.302		
Furukawa	405	151	0.616^b^	340	121	0.593^b^	745	272	0.365		
Horikawa	1830	690	0.6	1574	522	0.56	3404	1212	0.356		
Lee	908	324	0.61^b^	502	156	0.558^b^	1410	480	0.340		
Omori	1614	651	0.633	1045	381	0.6	2659	1032	0.388		
Sanghera	532	290	0.728^b^	349	188	0.732^b^	881	478	0.543		
Hu	1849	695^b^	0.613	1785	558^c^	0.559	3634	1253	0.345		
Tabara	493	162	0.591^b^	400	133	0.568^b^	893	295	0.330		
Chauhan	2466	1578^b^	0.8	2539	1505^c^	0.77	5005	3084	0.616		
Han	992	386	0.62	1005	327	0.57	1997	713	0.357		
Huang	443	134	0.541	229	64	0.483	672	198	0.295		
Lin	1529	532^b^	0.59	1439	420^c^	0.54	2968	952	0.321		
Ng	1481	485^b^	0.572	1530	433^c^	0.532	3011	918	0.305		
Ng	761	299^b^	0.627	632	216^c^	0.585	1393	515	0.370		
Ng	799	278^b^	0.59	1516	514^c^	0.582	2315	792	0.342		
Wu	424	144^b^	0.583	2786	899^c^	0.568	3210	1043	0.325		
Xiang	521	175^b^	0.579	721	203^c^	0.53	1242	377	0.304		
Tan	1541	433^b^	0.53	2196	617^c^	0.53	3737	1050	0.281		
Tan	1076	375^b^	0.59	2257	733^c^	0.57	3333	1108	0.332		
Tan	246	146^b^	0.77	364	199^c^	0.74	610	345	0.566		
**European Cohorts**											
Scott	2342	1011	0.649	2397	891	0.609	4739	1902	0.401	0.474	0.092
Sladek	2562	1440	0.746^b^	2878	1413	0.699^b^	5440	2853	0.524		
Steinthorsdottir	3776	1871	0.7	12361	5575	0.666	16137	7446	0.461		
Zeggini	1550	794	0.712^b^	2866	1393	0.694^b^	4416	2187	0.495		
Cauchi	2715	1453	0.729^b^	4255	2114	0.705^b^	6970	3597	0.512		
Cauchi	828	360	0.74^b^	952	367	0.699^b^	1780	727	0.408		
Cauchi	437	240	0.653^b^	676	331	0.626^b^	1113	571	0.513		
**Mexican Cohorts**											
Gamboa-Meléndez	1027	609	0.77	990	526	0.729	2017	1135	0.563	—	0.138

^a^ PAR calculated using ORs of 1.19, 1.21, and 1.28 for Asian, Caucasian, and Mexican cohorts, respectively;

^b^ risk allele frequency calculated from provided genotype incidences assuming Hardy-Weinberg equilibrium;

^c^ calculated assuming Hardy-Weinberg equilibrium: numbers with CC Genotype = p^2^n, where p is the risk allele frequency and n is the number of cases or controls

When calculating pooled ORs for the Asian and Caucasian cohorts included in subsequent PAR calculations, homozygous carriers of the CC genotype were observed to have a statistically significant increase in the odds of having T2DM compared to those with the CT or TT genotype: OR = 1.19, 95% CI: 1.06–1.33, p < 0.01; OR = 1.21, 95% CI: 1.13–1.30, p < 0.001, respectively. Results for the Asian cohorts were similar when all cohorts were used (above results) or when only the studies that reported explicit CC genotype numbers were used (OR = 1.20, 95% CI: 1.04–1.37, p < 0.01); therefore, results using all cohorts were used in the PAR calculation for the Asian population. As all included Caucasian cohorts reported explicit CC genotype numbers, no sub-analysis was necessary. No evidence of heterogeneity was observed in either group of cohorts (Asians: I^2^ = 0, p = 0.46; Caucasians: I^2^ = 10.4, p = 0.35). Using a random-effects model to calculate the pooled ORs resulted in similar results for both Asian and Caucasian cohorts (results not shown). Using reported data [22], homozygous carriers of the CC genotype in Mexican Mestizos were also observed to have a statistically significant increase in the odds of having T2DM: OR = 1.28, 95% CI: 1.08–1.53.

**Fig 1:**
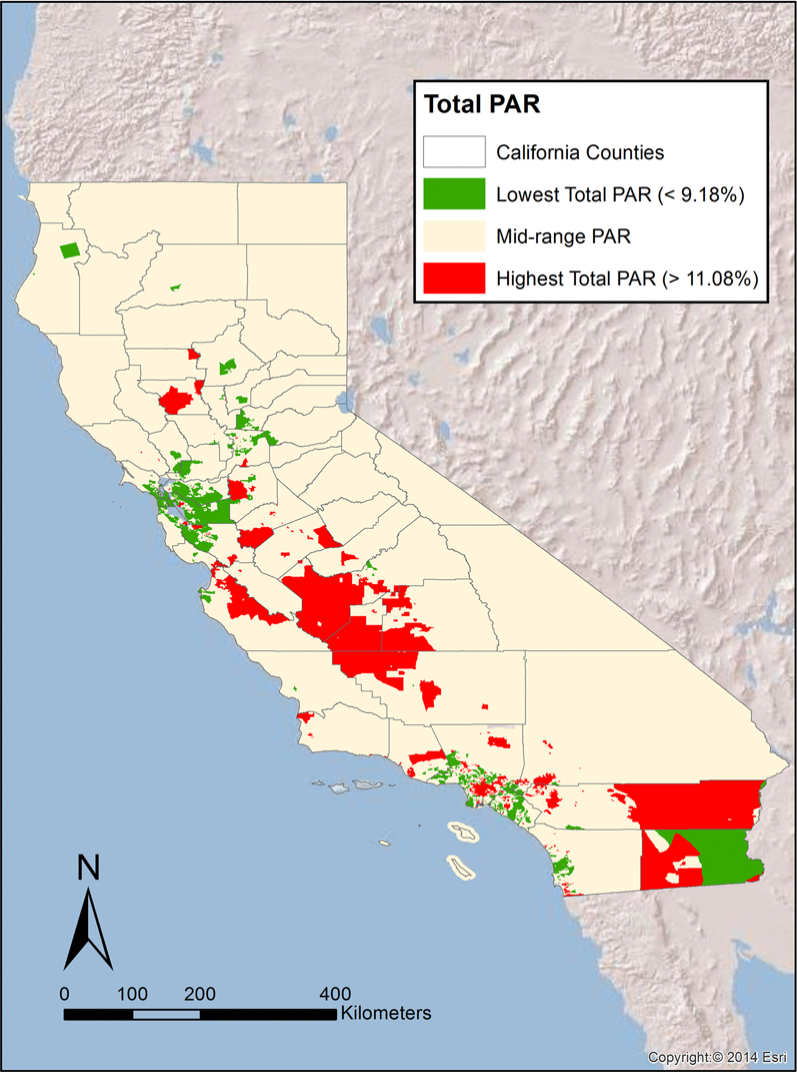
Geographic distribution of low and high PAR Census tracts across California. Census tracts in the green and red are those in the lowest and highest quintiles for Total PAR, respectively.

**Fig 2:**
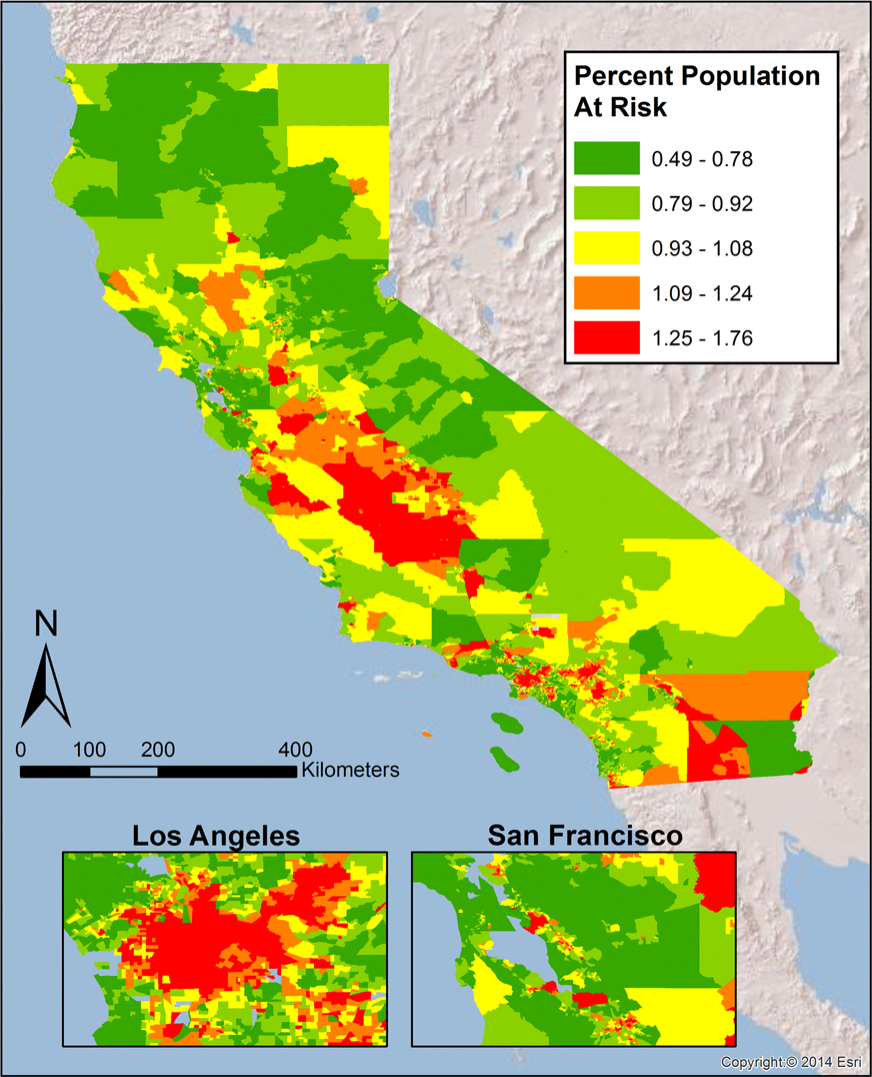
Percent of total population at increased risk of developing T2DM. Geographic distribution across the state of California for percent of population at increased risk of developing T2DM due to the rs13266634 single nucleotide polymorphism.

The genetic prevalence data and pooled ORs were used to calculate PAR values for the individual ethnic groups (Table 1). Mexican Mestizos had the highest PAR of the three ethnicities (0.138), more than double the risk in Asian-American populations (PAR = 0.065); Caucasians were observed to have a PAR of 0.092. PAR values for the three ethnicities were then combined with the census (tract level) data to generate PAR maps (categorized by quintiles). These PAR maps (S1–S4 Figs) present the geographic distribution across California of the proportion of T2DM cases due solely to the rs13266634 SNP in the SLC30A8 gene for individual ethnicities and the total population in aggregate. For example, when investigating the expected prevalence of T2DM due to the Asian-American population in California, the PAR is very low (> 1%) across the majority of the state (S1 Fig). This reflects that the small number of Asian-Americans living in rural portions of California contributes very little to the expected prevalent cases of T2DM in those areas. Conversely, the PAR due to solely to the Caucasian population in these areas is much higher (~6–9% prevalent cases of T2DM, S2 Fig), reflecting the larger percentage of the total population Caucasians represent in these areas, and thus the larger contribution that population makes to T2DM prevalence. The greatest values for T2DM PAR are located in areas where Mexican-Americans (of any race) make up a large portion of the population: the San Joaquin Valley, near and within Los Angeles (Inland Empire), and southern California (e.g., San Diego and Imperial and Riverside counties) (S3 Fig). As should be expected, the PAR values for the total population are highest in areas with a larger Mexican population and lowest in the regions with the greatest Asian-American populations (S4 Fig). This pattern is more discernable when only the lowest (<9.18) and highest (>11.08) quintiles of total PAR are mapped (Fig 1). Total PAR values can be additionally be combined with information on T2DM prevalence to estimate the percentage of the total population (not just percentage of T2DM cases) that are at increased risk of developing T2DM due to the rs13266634 SNP (Fig 2). The Centers for Disease Control estimate the age-adjusted percentage of people over the age of 20 with diagnosed diabetes (2010–2012) as 4.4% for Chinese, 13.0% for Asian Indians, 8.8% for other Asians, 7.6% for non-hispanic whites, and 13.9% for Mexican-Americans, and 13.2% for African-Americans [48]. Using the specific prevalence rates for individual Asian ethnic groups, a weighted average of 7.2% for the Asian population as a whole (based on the individual Ns from the Asian cohorts in this analysis) was calculated. Combining this prevalence data with census tract population figures for all ethnicities, an average of 0.98% of the population across census tracts is at increased risk of developing T2DM due to the CC risk genotype. This corresponds to approximately 414,000 Californians at risk (95% CI: 179,000–640,000).

As decisions regarding remediation and/or intervention strategies may be more likely made on larger geographic units than census tracts, counties that contained census tracts in the highest total PAR quintile (Q5 census tract) were identified (Table 2). In total, 1598 Q5 census tracts were identified, with approximately 24% of the total state population residing in those census tracts. A majority of California counties (32 counties) contained at least one Q5 census tract. Only two counties (Imperial and Monterey counties) had a majority of their population residing in Q5 census tracts (95% and 52%, respectively). However, half of the counties (16) had more than 25% of their population residing in Q5 census tracts, and 66% of counties (21) had more than 10% of the population in Q5 census tracts.

**Table 2:** Counties with at least one Census Tract in the highest quintile of Total PAR.

County	# Q5 Census Tracts^a^	Population of Q5 Census Tracts	Total County Population^b^	% County Population in Q5 Census Tract
Imperial	27	243917	256229	95.19
Monterey	34	284769	547350	52.03
Tulare	33	309791	625850	49.50
Kern	57	461007	1009155	45.68
Merced	18	149471	328035	45.57
Madera	7	85188	204339	41.69
San Benito	4	30285	74766	40.51
Kings	9	78545	194112	40.46
Colusa	2	10752	26680	40.30
San Bernardino	133	900444	2317432	38.86
Fresno	65	429636	1110640	38.68
Los Angeles	758	3792716	10431176	36.36
Ventura	43	322307	964413	33.42
Riverside	126	796176	2503008	31.81
Santa Cruz	10	96146	313820	30.64
Santa Barbara	16	153888	522385	29.46
Orange	78	623963	3367394	18.53
Stanislaus	15	109064	638912	17.07
Glenn	1	5266	33053	15.93
San Diego	83	559336	3565553	15.69
San Joaquin	18	97050	777986	12.47
Sutter	1	8183	106353	7.69
San Mateo	7	49375	760551	6.49
Alameda	24	82034	1431291	5.73
Santa Clara	19	107898	1954032	5.52
Contra Costa	7	44313	1047349	4.23
Sonoma	3	20487	530552	3.86
Yuba	1	6515	223305	2.92
Marin	1	6825	245102	2.78
Sacramento	4	23590	1387263	1.70
San Luis Obispo	1	3873	291604	1.33
Tuolumne	1	496	56009	0.89

^a^ Census tracts in the highest quintile of total PAR as identified in Fig 2.

^b^ Total population in county calculated as the sum of all census tracts in that county

## Discussion

The current analysis presents a predictive risk screening approach to identifying census tract locations of communities potentially at risk of developing chronic diseases due to genetic susceptibility factors. Through the mining of genome-wide association study databases, we were able to identify genes encoding proteins that rely upon metals for their catalytic activity, and their relationship to T2DM. A similar approach has been used previously: an environment-wide association study (EWAS) using survey-weighted logistic regression was conducted on the NHANES data from 1999–2006 to identify chemical exposures and nutrients that may be associated with T2DM [49]. They found statistically significant odds ratios for PCB170, hepatachlor epoxide, and the nutrients cis-beta-carotene, trans-beta-carotene, and gamma-tocopherol across more than one NHANES cohort.

We used prevalence information about the SLC30A8 gene polymorphism rs13266634 to perform a geographic and demographic-based predictive screening pilot focused on the State of California. In this pilot we identified census tracts with elevated PAR for developing T2DM based on the prevalence of rs13266634 in various human populations. Census tracts with a higher PAR will likely contain individuals who may respond more poorly to chemical exposures.

While this study highlights a method for incorporating information on markers of genetic susceptibility with data on the spatial distribution of potentially susceptible populations, there are important limitations that warrant discussion. This analysis used multiple studies [10,11,13,16,24–41] to investigate associations between the rs1326664 C/T polymorphism in the SLC30A8 gene and prevalence of T2DM in Asian and European populations, but only one study to characterize risk in Mexican populations [22]. Confidence in the Mexican PAR value may therefore be lower than PAR values calculated for the Caucasian and Asian populations as those values were calculated using pooled ORs. Additionally, the pooled European and Asian ORs and the single Mexican OR have not adjusted for possible confounders. By not including confounders in the present meta-analysis, it is possible that the raw ORs may not adequately account for the contributions of other environmental or behavioral components of T2DM risk. However, the majority of studies from which the individual cohorts were drawn did account for numerous confounders (e.g., age, sex, obesity), and the ORs (both allele- and genotype-specific) calculated in those studies remained statistically significant after adjustment. Therefore, it is likely that any pooled OR estimated via meta-analytical techniques from these studies would also remain statistically significant. Regardless, if this methodology were to be used in an actual risk screening effort, more rigorous meta-analytical techniques that do incorporate information on confounders should be considered.

Although the current analysis independently calculated pooled ORs from the Asian and European cohorts, African cohorts were not included as the reported association between the C/T polymorphism and T2DM was not statistically significant in a pooled analysis (OR: 1.20, 95% CI: 0.90–1.40) [8]. As a result, when calculating the ethnicity-specific and total PAR for California census tracts, the African-American populations in individual census tracts were excluded. Although the primary goal of the current analysis is a “proof-of-concept” for integrating multiple sources of genetic, spatial, and health effects data to characterize population-level risks, omission of the African-American populations limits the interpretability of the PAR mapping results. In census tracts with large African-American populations, the true total PAR may be different from the current results depending on CC frequencies in African-Americans and which OR was used in the PAR calculations.

In future analyses, the African-American population could be incorporated in one of three ways. The first method would be to simply incorporate elevated ORs for African cohorts (i.e., >1.0) ignoring statistical significance. A second approach would be to acknowledge that the increase in the pooled African OR is not statistically significant, and to include the African-American population numbers in the denominator when calculating the population-weighted total PAR (Eq 2). This would be equivalent to calculating a PAR for African-Americans using an OR equal to 1 (thus, the PAR would be calculated to be zero). However, this approach would give undue weight to the risks in other ethnicities as it considers the central estimate of risk for those populations while ignoring the observed, albeit non-statistically significant, increase in the central estimate of risk in the African cohorts. The third alternative would be to calculate the PAR values based on the lower limit of the 95% confidence interval for each ethnicity, substituting 1.0 for the African cohorts. This approach would represent the most conservative estimate of risk of T2DM due to the CC genotype, but would incorporate the risk in each ethnicity equivalently.

Another limitation in the current analysis is how ethnicity-specific information has been incorporated. First, smaller ethnic groups have been aggregated into larger sets for the purpose of calculating the PAR. For example, study populations that have been described as Asian in this analysis are made up of Han Chinese, Korean, Japanese, and Asian Indian populations. Grouping different, distinct ethnic groups, each with their individual risks, allele frequencies, and spatial distributions, most likely masks true patterns in genetic susceptibility. Disaggregated information is available on these populations on the census tract level from the Census Bureau, and could be incorporated in future analyses. However, consideration of the smaller ethnic groupings may result in issues discussed above, namely findings of non-statistically significant risks for some smaller ethnic groups. Another assumption of this analysis is that the PAR calculated from the Asian, European, and Mexican Mestizos cohorts are representative of Asian, Caucasian, and Mexican-American populations in the United States. This may be true for populations of recent immigrants, but established ethnic populations could be sufficiently different such that the PARs calculated are not representative of the true risk for multiple reasons, especially differing risk allele frequencies. In order to minimize the uncertainty in whether this assumption is true, studies investigating the risk in these populations actually living in the United States would need to be identified and incorporated into the analysis. Lastly, race and Hispanic origin are considered separate concepts by the Census Bureau, and data regarding these self-reported identities are collected independently [50]. In the current analysis, the Mexican-American population category is listed as “Mexican—of any race”, meaning that those self-identifying as Mexican can also self-identify as Caucasian, African-American, etc. This is a source of uncertainty as it is likely that some unknown proportion of the population in individual census tracts have been counted twice (as both Caucasian and Mexican) in PAR calculations.

A natural extension of the current study is to investigate whether spatial patterns of T2DM risk due to genetic susceptibility correlate with spatial patterns of environmental pollutants, other determinants of disease, and T2DM prevalence. Future studies could locate spatially-resolved data on contamination from sources such as EPA’s Toxic Release Inventory (http://www2.epa.gov/toxics-release-inventory-tri-program) or National Priorities List (http://www.epa.gov/superfund/sites/npl/), and compare the clustering of high levels of contamination with the spatial patterns of total PAR distribution. Other sources of vulnerability to disease (poverty, socioeconomic status) could be incorporated into future analysis using currently available tools such as the CDC’s Social Vulnerability Index (www.svi.cdc.gov). Publically available information on T2DM prevalence is most likely only available on the county level. This discrepancy in spatial resolution between T2DM data and contaminant and PAR data would be a challenge in determining if census tracts with increased genetic susceptibility and environmental contamination also are observed to have increased T2DM rates.

## Conclusions

This study describes a method for performing predictive risk screening to identify census tracts which may contain populations with increased genetic susceptibility to developing T2DM. In our pilot project, we have identified several census tracts within the State of California where potentially susceptible individuals live, suggesting these are potential areas where there may be environmental justice concerns. Concerns regarding environmental justice would be more pressing if the areas with elevated genetic susceptibility were collocated with areas with increased exposure to environmental pollutants also associated with T2DM risk.

This methodology potentially enables risk managers and policymakers to prioritize sites for cleanup and regulatory action, as well as help inform local decisions about commercial and industrial siting, zoning, and land use. In addition, this predictive screening approach may facilitate the problem formulation step of future risk assessments by identifying possible associations between disease endpoints and chemical exposures, and estimating the size of potentially susceptible populations across the United States. This will also facilitate environmental justice screening by allowing risk assessors and risk managers to identify communities which may bear a disproportionate risk due to their demographics and genetic susceptibility.

## Supporting Information

S1 Fig Geographic distribution of PAR for Asian-Americans across California. (PDF)

S2 Fig Geographic distribution of PAR for Caucasians across California. (PDF)

S3 Fig Geographic distribution of PAR for Mexican-Americans across California. (PDF)

S4 Fig Geographic distribution of Total PAR across California. Total PAR is the subgroup population-weighted average PAR for the total population in each census tract (i.e., the sum of the Asian-American, Caucasians, and Mexican-American populations, see Methods).
(PDF)
