# Supplementary figures and images for: Can Data Science Inform Environmental Justice and Community Risk Screening for Type 2 Diabetes?
Source: PLoS One. 2015 Apr 14;10(4):e0121855. doi: 10.1371/journal.pone.0121855 (PMC4396977; doi:10.1371/journal.pone.0121855)

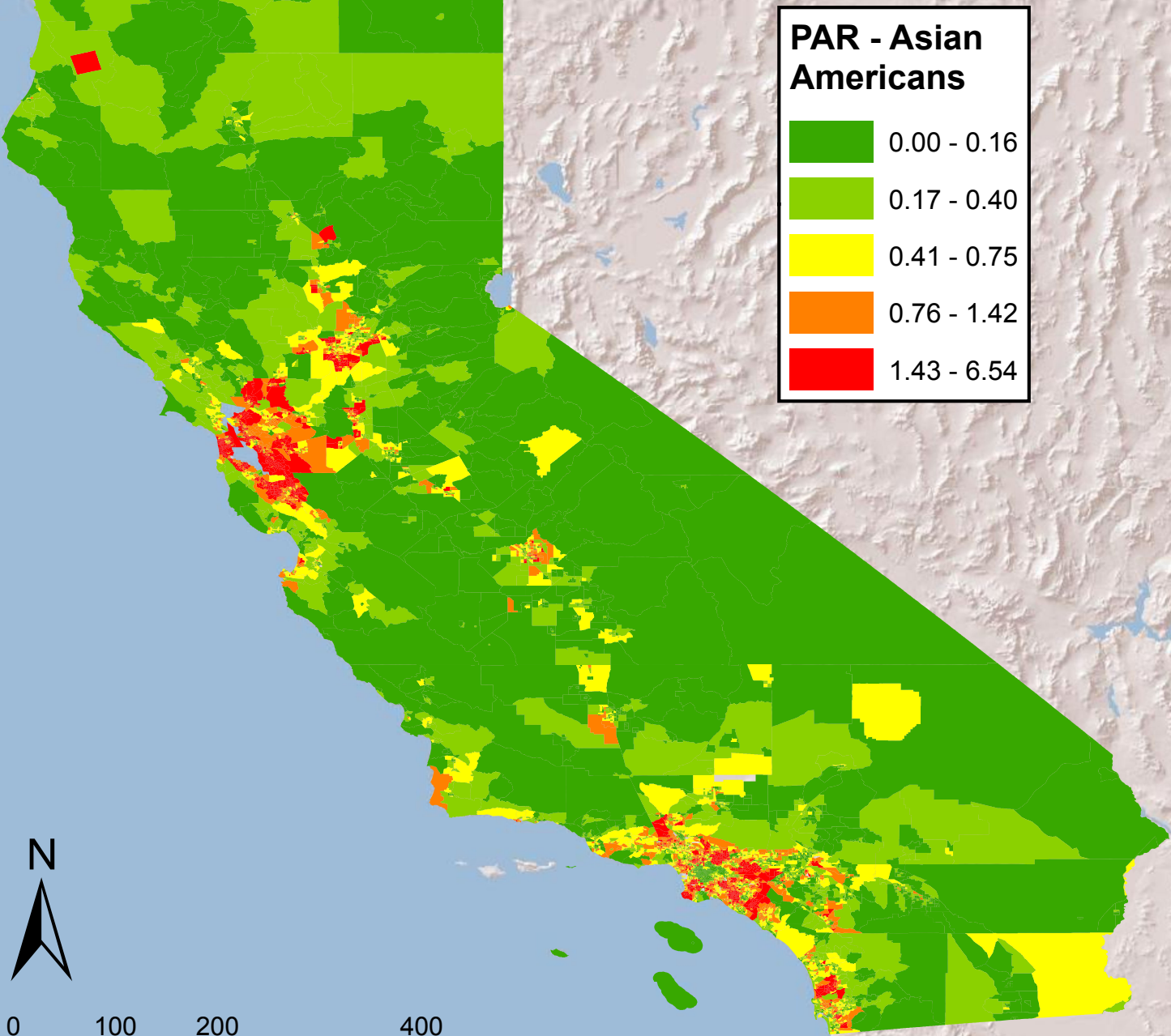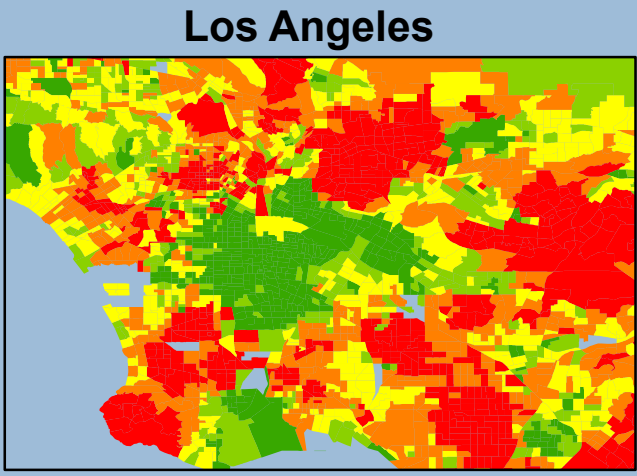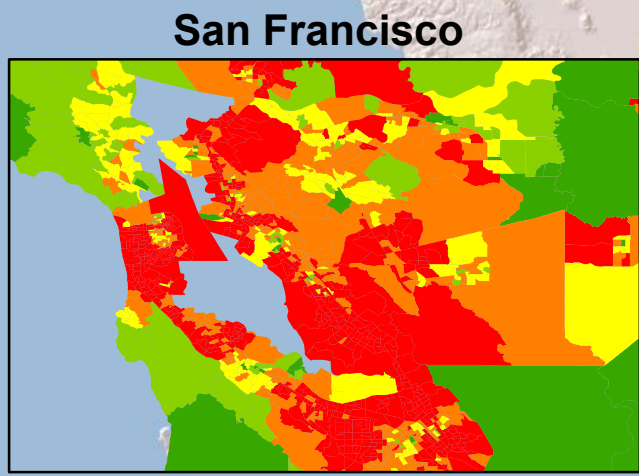

Supplement: S1 Fig — (PDF) [file pone.0121855.s001.pdf]

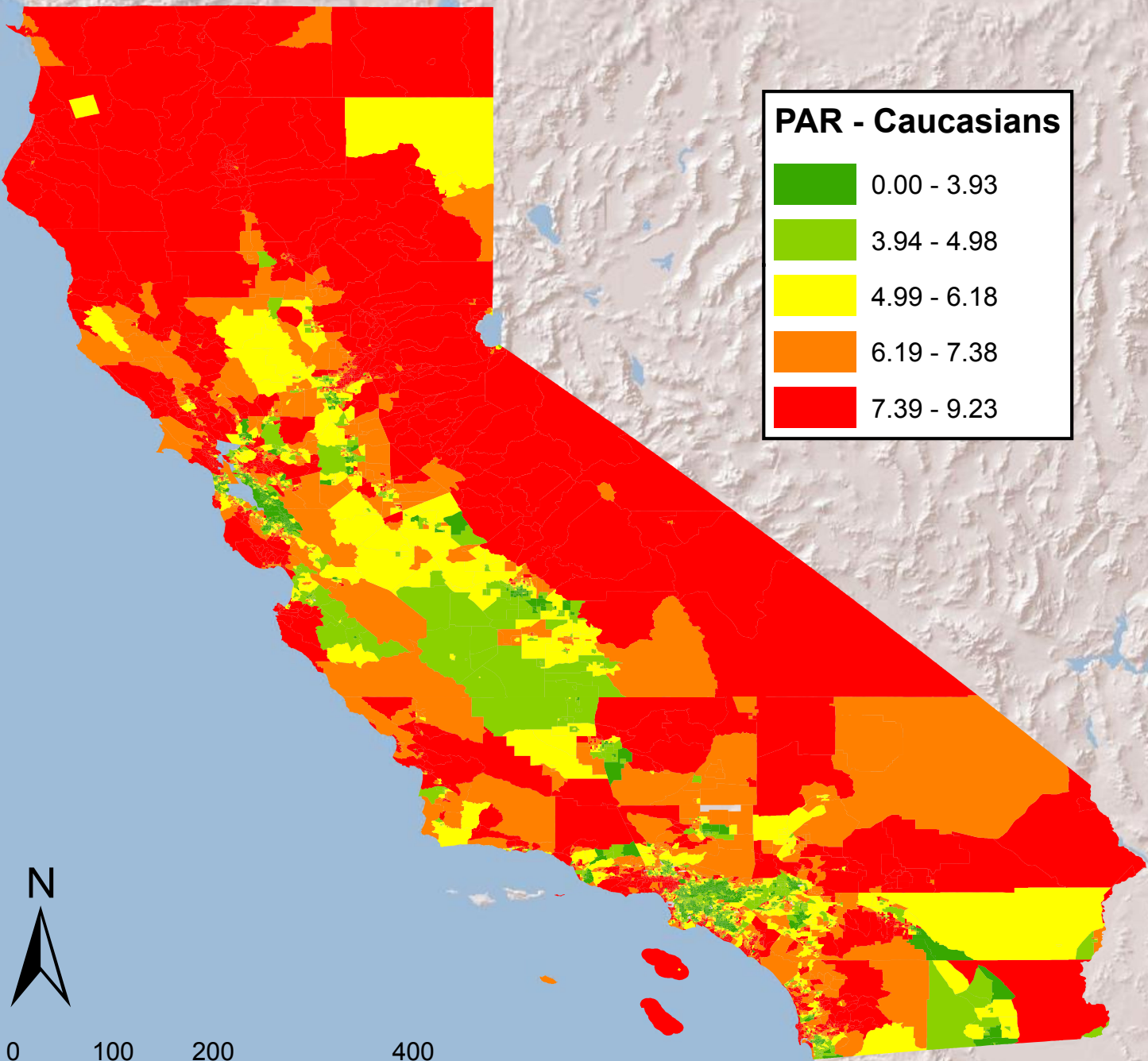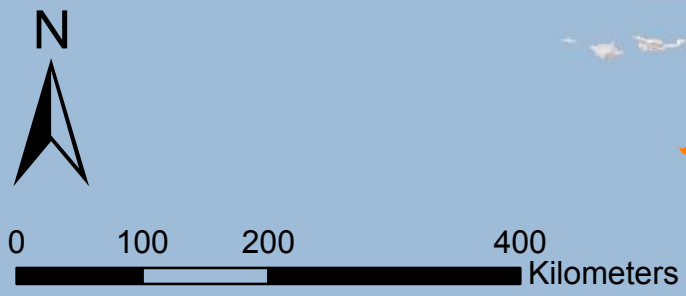

**Los Angeles**

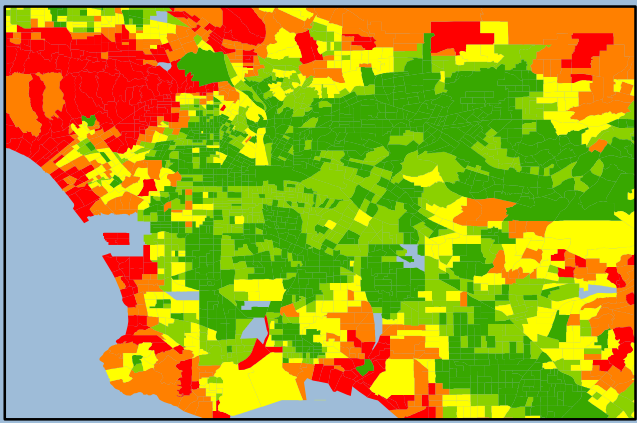

**San Francisco**

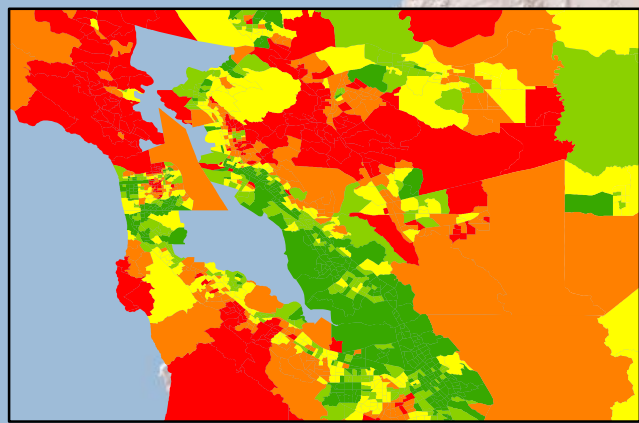

Supplement: S2 Fig — (PDF) [file pone.0121855.s002.pdf]

**PAR - Mexican Americans**

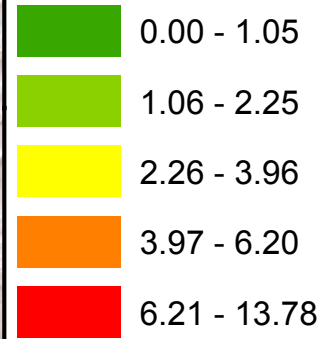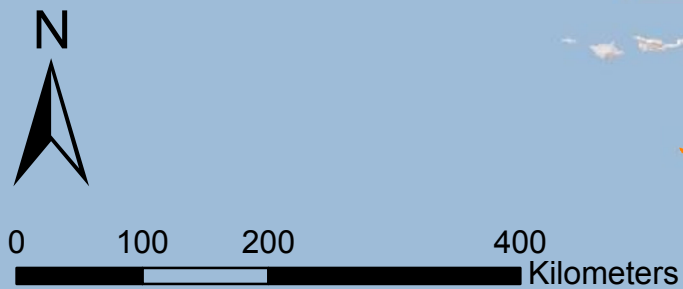

**Los Angeles**

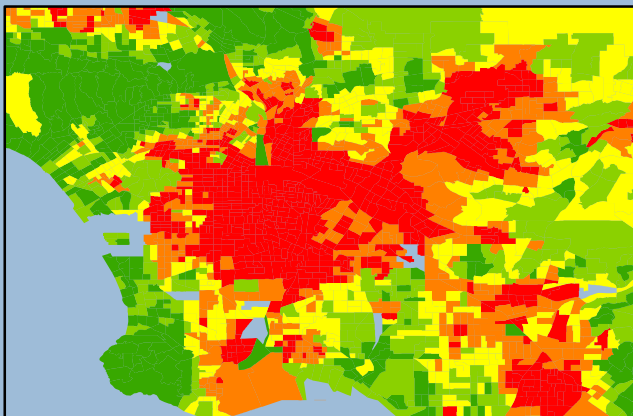

**San Francisco**

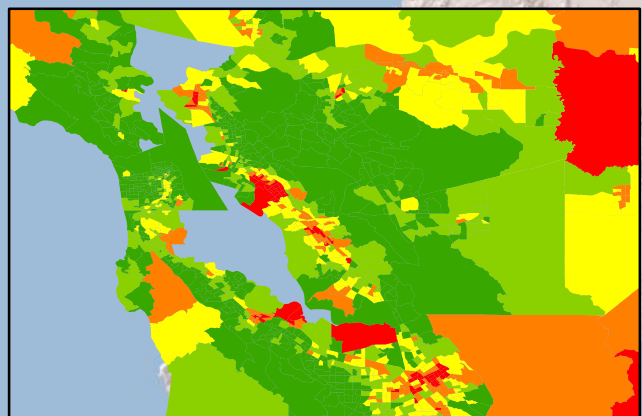

Supplement: S3 Fig — (PDF) [file pone.0121855.s003.pdf]

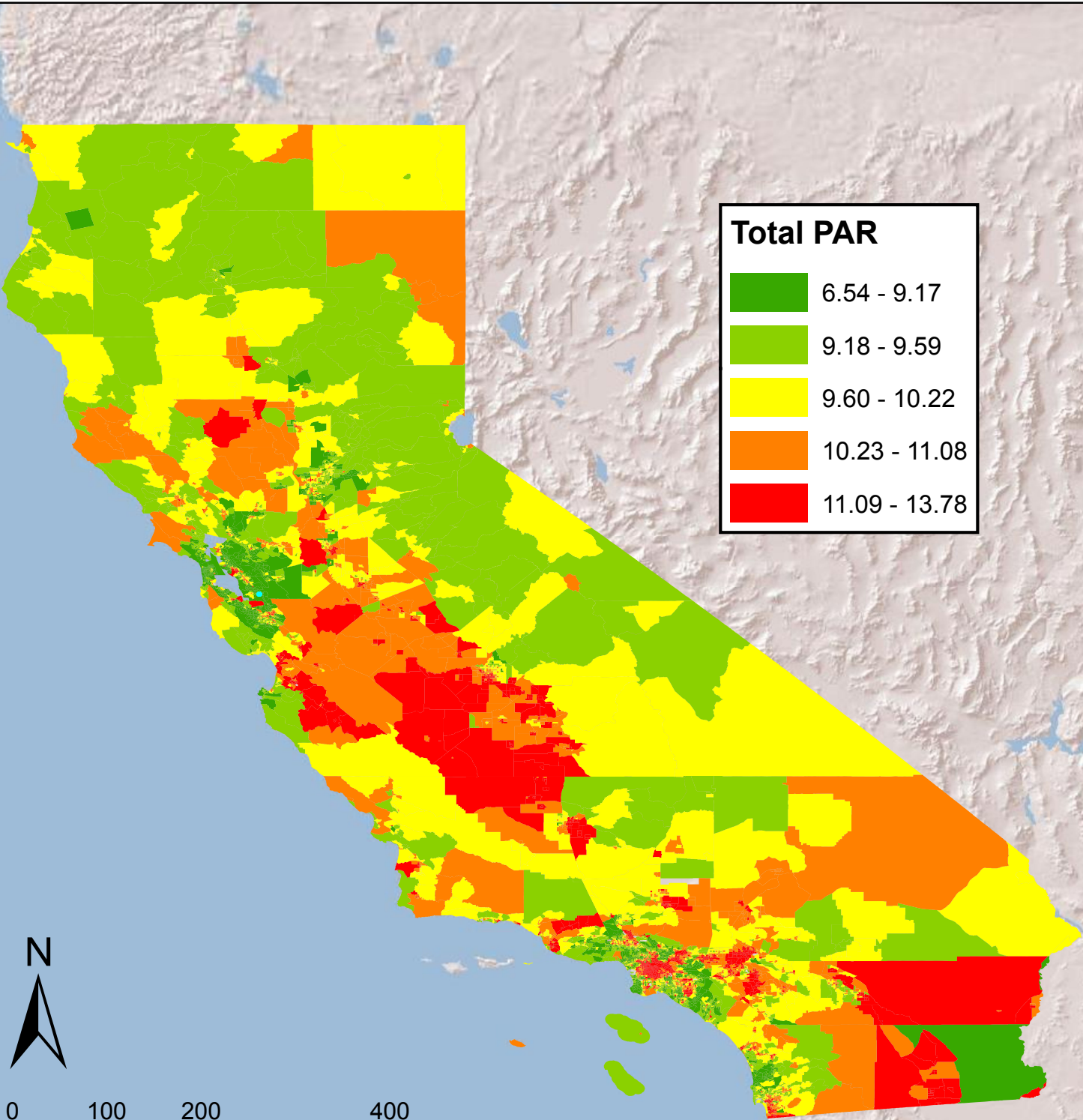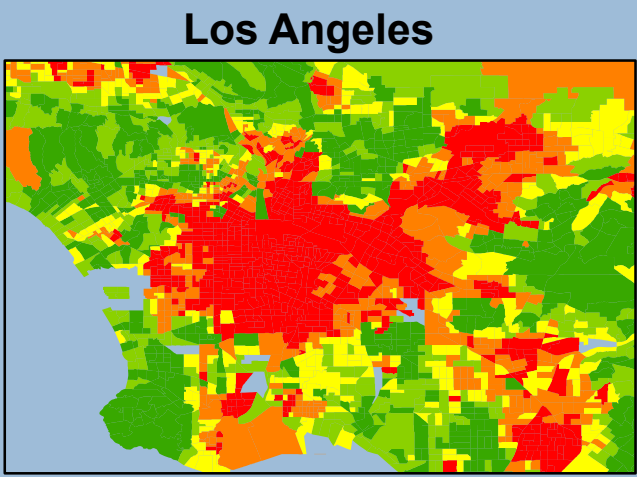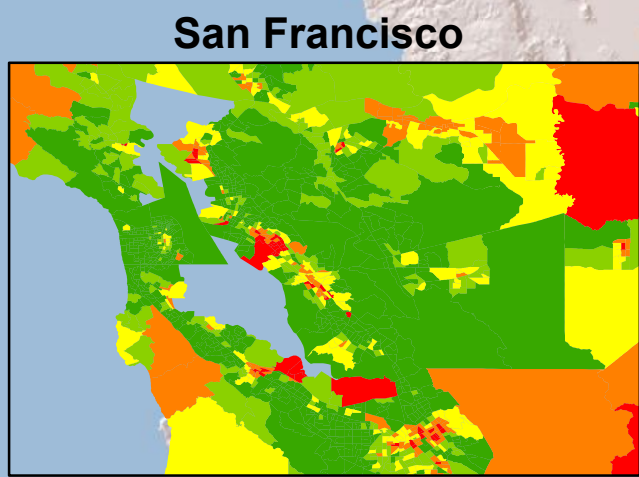

Supplement: S4 Fig — Total PAR is the subgroup population-weighted average PAR for the total population in each census tract (i.e., the sum of the Asian-American, Caucasians, and Mexican-American populations, see Methods). (PDF) [file pone.0121855.s004.pdf]
